# An audit to review the extent to which appropriate preconception advice is given to women being discharged from mental health wards on psychotropic medication, in line with NICE guidelines

**DOI:** 10.1192/bjo.2021.282

**Published:** 2021-06-18

**Authors:** Helen Muskett, Kirsty Bradley, Lauren Stott, William Moreton, Sarah Jones

**Affiliations:** 1 Pennine Care NHS Foundation Trust; 2 Laureate House, Wythenshawe Hospital; 3 Manchester Foundation Trust

## Abstract

**Aims:**

The release of the Cumberlege Report in 2020 served as a reminder of the importance of informed consent for women when they are started on treatment that may affect their fertility or future pregnancies.

Our aim was to evaluate current performance with regards to advice given to women of childbearing age around contraception, impacts of psychotropic medication on fertility and future pregnancies, and availability of preconception counselling.

**Method:**

Standard identified as NICE Guideline 192 (Antenatal and Postnatal Mental Health), sections 1.2 and 1.4.

60 female inpatients were selected by looking at the most recent discharges prior to 03/11/2020 from 3 local acute adult wards. All females aged between 18 and 48 years were included.

Electronic notes were reviewed for each patient. The discharge summary and last four ward round entries were reviewed, then key-word search of the patients’ records was performed using the terms “pregnan*”, “conception”, “contraception”, and “fertility”.

The following information for each patient was documented in a spreadsheet:

Discharge medication

Is there any discussion or advice around contraception?

Have women taking antipsychotic medication been given advice regarding the possible impact on fertility?

Has the potential impact of psychotropic medication on a future pregnancy been discussed?

Has advice been given about the availability of preconception counselling should they plan a pregnancy in future?

**Result:**

On discharge, a total of 33 women were taking one or more antipsychotics and 14 were prescribed a benzodiazepine. 24 women were discharged with antidepressants and 10 women were using a mood stabilising agent. 8 women were discharged without any psychotropic medication.

Overall, 4 women received advice about contraception, and a further 8 women were already using contraception. The impact of taking an antipsychotic on fertility was not discussed with any patient. No women were advised about pre-conception counselling. The impact of taking psychotropic medication on a future pregnancy was discussed with one woman.

**Conclusion:**

Current practice falls well below the standard set by NICE. Opportunities to inform women are being missed, and this has implications for the wellbeing of the patient and, potentially, future children.

Action plan;

Present findings at teaching.

Deliver local teaching covering preconception counselling and the role of adult mental health teams when managing women of childbearing age.

Produce a poster for inpatients wards and an information leaflet for women of childbearing age to aid with discussions.

Create a poster for doctors’ offices to remind about NICE standards and documentation.

Re-audit in 6 months.

